# Her Heart's Mistake

**Published:** 1889-08-17

**Authors:** E. D. Cumming

**Affiliations:** Author of "An Ordeal by Silence"


*(To be continued.)*

### Her Heart's Mistake.

By E. D. Cumming, Author of "An Ordeal by Silence."

*(Continued from page 299.)*

### Chapter XVIII.—"Unheedful Vows may Heedfully be Broken."

"A more providential escape I never heard of," said Mrs. Macdonald, the following evening at the club. "Not one man in ten thousand would have been equal to the occasion. I wouldn't have believed Mr. Warren had it in him."

"I can't close my eyes without seeing the horrid thing on the Doctor's wrist," said Mrs. Arkwright, with a genuine shudder.

"It was no wonder Kate fainted," said Mrs. Strachan.

"What a lucky accident it was that Mr. Warren happened to be there. The Doctor owes him his life."

"Mr. Warren deserves the utmost credit for his readiness of resource," said Mrs. Tenterdale, "but to my mind it would have been an unlucky accident if he had *not* been there."

"Why?" asked Mrs. Pringle, shortly.

"My dear Mrs. Pringle, the man's never out of the house. Kate makes no secret of it."

"Why should she?" enquired Mrs. Pringle.

Mrs. Tenterdale looked at her in surprise.

"I don't think that a girl in her position should encourage one gentleman visitor to go there so frequently. It's not fair to Captain Bairdsley, particularly while he is away."

Mrs. Pringle was about to reply, when Mrs. Arkwright, who was leaning over the verandah railing, exclaimed, meaningly:

"There they are; together again!"

She pointed to the carriage in which Dr. Harrison, Kate, and Warren were driving slowly down the Mall. It was rather gratuitous to say "together again," inasmuch as she had never seen them alone together at any time; but there seemed to be the germ of an inviting scrap of scandal in what Mrs. Tenterdale had let fall, and Mrs. Arkwright was not the woman to let it perish for lack of nurturing. Meerut was very dull and idle just now, and Scandal is Idleness' pet child.

When Warren left the house on the previous evening, he did so, knowing that Kate Harrison loved him; and for a few hours he was undecided how to act. She was engaged to marry George Bairdsley, and he, Richard Warren, had supplanted him in her affections. He was a high principled man, and the thought that he had stolen a march upon the absent lover was horrible to him; and he felt that no course was open to him but to leave the station at once. But as he tossed from side to side on his sleepless bed he began to take a different view. Kate Harrison was before all things sincere and essentially honest. Her eyes had unwittingly confessed her love for himself; it was therefore impossible that she had given her heart to her affianced husband. He could not but recall her father's words, that he was willing that her marriage should be delayed, in order that she should be perfectly sure of her own mind. The speech suggested that the Doctor had had his doubts on the subject at one time.

"I will know the truth from her own lips," he said to himself, "and if she confesses that she has mistaken her feelings towards him I will claim my rights."

He went, the next afternoon, to the corner bungalow, but found Dr. Harrison with her, on the point of starting for a drive. He was invited to accompany them, and he went; he had cast his scruples to the wind, and was recklessly abandoning himself to fate. If anything had been wanted to confirm Kate's unspoken admission of the night before her demeanour during the drive did it. She was silent and nervous, and he caught her eyes from time to time and saw in them an expression he understood only too well. The fortress was his own, but he was to fight for it. Be it so; he was not the man to shrink from such a contest, for such a prize; no power on earth should turn him from his purpose now.

The evening was cloudy, and a light wind made the atmosphere cool and pleasant; but that hour in the carriage seemed interminable to Kate. Warren talked commonplaces in even conventional tones, in no way differing from his ordinary voice; but she knew that he had read the secret of her inmost soul last night; that he had received with his own her love for him at the very moment of its birth; and she was waiting in suspense for the time when they should be alone. It came as soon as they returned to the house, for the Doctor remained in the carriage briefly explaining that he was going up to the hospital before dinner. Kate saw him drive away, and went into the verandah, followed by Warren; she sat down, but he remained standing before her, resting his hand on the back of a chair. She was as pale as death, but she had resolved to do what she felt to be right, and was prepared. She was engaged to George, and would be faithful to him at any cost. "For ever," had been her last farewell to him, and though her love for this man ousted all feeling but sorrow and remorse, she would remain true to her promise.

"You know what you told me last night?" said Warren, in a low voice.

"I told you nothing," she answered, trembling violently.

"You *said* nothing, Kate!" he exclaimed, suddenly breaking loose from his self-restraint. "Why need you trifle with me now? You cannot retract what you said without words last night! You——"

She rose from her seat and tried to look him in the face as she cut him short.

"You forget yourself, Mr. Warren; I am engaged to marry Captain Bairdsley."

But she spoke unsteadily and could not meet the gaze fixed upon her.

"I know it," he said sternly. "Had I not known it I should have spoken ere now. I did not come to hear you speak of him; I came to ask if you will deny that you love me."

"How dare you ask me that?" she cried, while the blood rushed to her face, and her heart throbbed as though it would burst. "How dare you come —— "

"Kate!"

She sank into a chair and covered her face with her hands, sobbing bitterly in her distress. She could not deny her love, and the paltry attempt she had made to carry matters with a high hand had been a transparent failure. He came nearer to her side, and continued:

"I will not ask you again to deny it, Kate. If you care for me, you never loved Bairdsley."

"I do!" she exclaimed almost desperately, as though trying to convince herself what she now knew to be false was true.

"I say you did not, that you do not. Listen to me, Kate!" he cried. "I will not leave this house until you tell me that you will set aside the promise you have made to this man. He has no right to it when I have your love. You shall tell him the truth and claim your freedom."

"I cannot; I dare not," she sobbed.

"I say you must, and you shall. What right have you to throw away your own happiness and blight his by such a mockery? Will you give him your empty hand? Do you know what a loveless marriage means, Kate? You cannot reclaim your promise!"

"Oh, Dick, Dick." She could look him in the face now; she could be honest with him, and beg him not tempt her to break her word.

"My darling, I only ask you to do what is just and right. You have made a terrible mistake, but have discovered it in time. You will not condemn yourself, and me, to life-long misery for this promise you have given?"

"I cannot break my word. O, Dick, don't ask me to be false to my word!"

"I do not ask you to break it. I ask you, no, I command you, to reclaim it from this man. Write to him, and tell him that you have made this mistake, and how you have come to realise that you cannot give him what you ought. No man worthy of the name will hold you to such a bond. He may reproach you, and punish your error thus, but what is that to the alternative?"

She could not answer; she knew he was right, and that in justice to Bairdsley she ought to tell him the truth. But how could she explain that she never found out that her heart had betrayed her until another taught it how to love? That parting with him in the railway carriage rose before her as vividly as though it had taken place that day. "For ever," she had said, believing, yes, honestly believing, that she loved him with all her soul.

"Will you do this, Kate?" asked Warren gently, "or will you go to this man with a lie upon your lips, and destroy not only your own life's happiness, but his and mine? Is it a little thing to do this? Is it so great a task to confess that you have made a mistake? Have you the courage to sacrifice your love, but not to admit you have been misled by your feelings?"

Still she could not answer. She sat with her hands tightly clasped, staring at the floor with dry, tearless eyes that saw nothing. Warren laid his hand upon her arm and pleaded his cause with greater earnestness, though his voice was quivering with emotion.

"Kate, have you the right to refuse this to the man you love? Can you tell me that you consider yourself irrevocably bound to another by a promise which should never have been made? In the name of mercy, do not tell me this!"

"Give me time, Dick," she answered, imploringly. "He trusts me so implicitly, and, oh! I did think that I loved him. I only want to do what is right, and I cannot break my word, though I love you as I never cared for him. Give me time to think, Dick."

"I will say no more, Kate; remember that it lies with you to shape the course of three lives. I *know* that you will do what is right."

He took her hand and kissed it, and left the house without another word.

Kate fled to her own room, and threw herself on the bed in an agony of love and remorse. She saw how she had deceived herself now; she had mistaken admiration for love. She had never looked for Bairdsley's coming as she looked for Warren's; when the former was at her side she was content that the rest of the world should pass them by and leave him to her, undisturbed. But Warren's absence left a blank, a void which she never understood until last night; she had deluded herself into believing that she welcomed him for her father's sake, but now she knew that his presence in the house, the knowledge that he was near, had a charm which lay far deeper than the pleasure that Bairdsley's companionship had given her. She had thought that similarity of taste was love; that because they had some things in common, their more subtle sympathies must of necessity be akin. And she had lived this benighted life, asking nothing more, because she knew no more, and thought that it was love. She had watched her little star, thinking it the moon, until the greater clearer light had arisen, to show her how terribly she had erred.

For three months she had been engaged to Bairdsley, and while he had been away she had written to him tenderly, confidingly, but ever with a hungry, nameless craving she could not banish. She had struck a false note so like the true one, that he had never detected the difference, and was at this moment living in the dream from which she must awaken him. She *must* tell him the truth, and beg him to release her; he was all that was kind and generous, and he would not hold her to her promise against her will. But how could she sit down and write such a letter? and how could she ever face him again? There was a hard task before her as punishment, but what was that compared to the misery she must inflict upon him? No shadow of doubt as to her constancy had ever crossed his mind, except that momentary jealousy aroused by her reference to Warren. To Warren! The memory of those letters she had written cut her like a knife as she lay face downwards on the pillow sobbing in her wretchedness.

Her father came in to learn what had occurred, for the watchful servants had told him that she was weeping. He had no suspicion of the truth, and, sitting on the bed beside her, listened silently to her tearful confession.

"It is well that you should have found it out before it was too late, dear," he said; "but your course is clear. You must write to him and tell him openly and honestly why you cannot keep your word."

"I will not withdraw from my promise unless he consents. I have given it, and I will abide by it."

"He is not the man I take him to be if he refuses. You need not fear that, Kate. Only be honest with him, and tell him the truth unreservedly. He has a right to know everything."

He could not comfort her, and presently left the room to eat his dinner alone, for Kate had worked herself into a state of agitation which obliged her to remain in her own chamber. Exhausted with her troubles she fell asleep at length, and only awoke at eleven o'clock to find the house in darkness and silence, save for the low crooning song of her ayah in the back verandah. She arose calm and determined, and after washing her face and hands and rearranging her disordered dress sat down to write to Bairdsley. She hardly knew what she had written when she signed her name at the end; but she felt that if bitter humiliation and sincere regret could buy forgiveness for the wrong she had done him he could not refuse to grant it her.

